# Genome-Wide Identification of the MYB Family in *Morus atropurpurea* and Functional Characterization of *MaDIV* for Its Possible Involvement in Anthocyanin Biosynthesis

**DOI:** 10.3390/genes17060702

**Published:** 2026-06-17

**Authors:** Xuefei Chen, Yixin Liang, Xingxing Liu, Baozhong Zhu, Chengli Zhou, Wei Fan, Aichun Zhao

**Affiliations:** State Key Laboratory of Resource Insects, Institute of Sericulture and Systems Biology, Southwest University, Chongqing 400715, China; faychan@yeah.net (X.C.); onejiuyue@126.com (Y.L.); liuxingxing20@163.com (X.L.); zbz98@foxmail.com (B.Z.); 15775278386@163.com (C.Z.); fanwei2034@163.com (W.F.)

**Keywords:** *Morus atropurpurea*, *MYB*, anthocyanin biosynthesis, *MaDIV*

## Abstract

**Background**: Anthocyanin biosynthesis is tightly controlled by MYB transcription factors, yet the role of repressors, particularly those in the DIVARICATA-like (DIV) subfamily, remains poorly characterized. **Methods**: A genome-wide identification of MYB family members was performed in the mulberry (*Morus atropurpurea*) genome using a hidden Markov model and BLAST-based searches. Putative MYB genes were phylogenetically classified, and their expression profiles were analyzed across three fruit developmental stages. A DIV-like R2R3-MYB candidate, *MaDIV*, was functionally characterized via subcellular localization, quantitative real-time PCR, and heterologous overexpression in tobacco. **Results**: A total of 145 *MaMYB* genes were identified and classified into 31 distinct subfamilies. *MaDIV* expression showed a progressive decline during fruit ripening, which significantly correlated with increasing anthocyanin accumulation. Heterologous overexpression of *MaDIV* in tobacco led to a 42% reduction in floral anthocyanin content compared with wild-type plants. Concomitantly, the expression of the key anthocyanin biosynthetic gene *NtDFR* was strongly suppressed, whereas the flavonol synthase gene *NtFLS1* was significantly upregulated. **Conclusions**: These findings point to a possible involvement of *MaDIV* in the regulation of anthocyanin biosynthesis and provide preliminary evidence for the functional diversification of the DIV-like MYB subfamily in plants. The results contribute to a better understanding of the transcriptional control of fruit pigmentation in mulberry and related species.

## 1. Introduction

Anthocyanins, a major class of flavonoids, are responsible for the red, purple, and blue hues in numerous plant tissues and play critical ecological roles in pollinator attraction and seed dispersal [1,2,3]. Beyond their visual and ecological functions, these pigments are increasingly valued for their health benefits, including potent antioxidant activity and associations with reduced risks of chronic diseases, which enhance the nutritional and economic profile of crops [4]. The biosynthesis of anthocyanins proceeds through the flavonoid branch of the phenylpropanoid pathway, a conserved metabolic route that is predominantly regulated at the transcriptional level [5]. A central regulatory module involves the MYB-bHLH-WD40 (MBW) protein complex, which spatiotemporally controls pigment accumulation [6]. Within this complex, MYB transcription factors (TFs) are pivotal determinants, functioning as either potent activators or repressors to fine-tune anthocyanin production. Key structural genes in this pathway include *phenylalanine ammonia-lyase* (*PAL*), *cinnamate 4-hydroxylase* (*C4H*), *chalcone synthase* (*CHS*), *chalcone isomerase* (*CHI*), *flavanone 3-hydroxylase* (*F3H*), *dihydroflavonol 4-reductase* (*DFR*), and *anthocyanidin synthase* (*ANS*), which are coordinately regulated by MYB transcription factors [7].

Extensive research has characterized various R2R3-MYB activators, such as *MYB10* in strawberry and *MYBPA1* in blueberry, which enhance anthocyanin synthesis by binding to promoters of biosynthetic genes like *CHS* and *DFR* [8,9,10]. Conversely, repressor MYBs, including *MdMYB16* in apple and *VvMYBC2-L1* in grape, provide crucial negative regulation to prevent overaccumulation or ectopic pigmentation through mechanisms such as competition with activators or formation of repressive MBW complexes [11,12,13,14].

MYB proteins are categorized into subfamilies based on their repeat domains, with the R2R3-MYB group being the most prevalent and extensively studied in the context of anthocyanin regulation [15,16,17]. A notable subgroup within this class is the R-R-type, exemplified by DIVARICATA (DIV). Originally implicated in developmental processes like floral patterning and seed germination [18,19], emerging evidence suggests that DIV-like TFs also participate in specialized metabolism. For instance, *DIVARICATA1* promotes capsaicinoid biosynthesis in pepper [20], and *DIVARICATA3* modulates flavonoid and carotenoid accumulation in Oncidium [21].

In mulberry (*Morus atropurpurea*), several MYB activators of anthocyanin have been reported, but systematic genome-wide identification of the MYB family and functional characterization of repressive members, especially those belonging to the DIVARICATA-like subfamily, remain limited. Although DIV-like MYBs are primarily known for developmental roles, their involvement in anthocyanin repression has not been established in fruit crops. Mulberry is an economically significant species valued for its anthocyanin-rich fruits [22], yet the regulatory mechanisms governing pigment accumulation, especially those mediated by transcriptional repressors, are not fully elucidated.

In this study, we aimed to systematically identify MYB family members in mulberry, screen for candidates negatively correlated with anthocyanin accumulation, and functionally characterize a DIV-like MYB in anthocyanin regulation. Here, we report the identification and functional characterization of a DIV-like R2R3-MYB transcription factor, *MaDIV*. Using a combination of genomic, phylogenetic, and molecular approaches, we observed a negative correlation between *MaDIV* expression and anthocyanin content during fruit ripening. Heterologous overexpression in tobacco supported its potential role in regulating anthocyanin biosynthesis. These findings suggest that *MaDIV* may be involved in the regulation of anthocyanin accumulation in mulberry and provide preliminary evidence for the functional diversification of the DIV-like MYB subfamily, offering new insights into the complex regulatory networks governing pigmentation in fruits.

## 2. Materials and Methods

### 2.1. Plant Materials and Growth Conditions

Mulberry ZS5801 (*Morus atropurpurea*) fruits were collected from the Chongqing Sericulture Science and Technology Research Institute and the State Key Laboratory of Resource Insects of Southwest University, where mulberry trees were cultivated under standard field conditions with optimal agronomic management. Fruits at three distinct developmental stages were harvested: S1 (immature, green), S2 (color-turning, red), and S3 (fully ripe, purple). For each stage, fruits were pooled from at least five individual trees to constitute one biological replicate, with three independent biological replicates collected. All samples were immediately frozen in liquid nitrogen and stored at −80 °C until RNA extraction and anthocyanin content measurement. Transcriptome sequencing data for the above materials have been released in previous studies [23].

Tobacco (*Nicotiana tabacum*) plants were generated as previously described with modifications [24]. Tobacco seeds were surface-sterilized with 75% (*v*/*v*) ethanol for 1 min and 2% (*v*/*v*) sodium hypochlorite for 10 min, followed by five rinses with sterile distilled water. The sterilized seeds were sown on solid Murashige and Skoog (MS) medium and grown in a controlled growth chamber at 25 °C under a 16 h light/8 h dark photoperiod for subsequent transformation.

### 2.2. RNA Extraction and cDNA Synthesis

Total RNA was extracted from frozen plant tissues using RNAiso™ Plus (Takara Bio, Beijing, China) according to the manufacturer’s instructions. Briefly, approximately 100 mg of powdered tissue was homogenized in 1 mL of RNAiso™ Plus. The homogenate was mixed with 0.2 volumes of chloroform (Sigma-Aldrich, St. Louis, MO, USA), centrifuged, and the aqueous phase was collected. RNA was precipitated with an equal volume of isopropanol, washed with 75% ethanol, and dissolved in 50 μL of RNase-free water. RNA concentration and purity were determined using a NanoDrop™ 2000 spectrophotometer (Thermo Fisher Scientific, Waltham, MA, USA). Genomic DNA was removed using the gDNA Eraser, and first-strand cDNA was synthesized from 1 μg of total RNA using the PrimeScript™ RT Reagent Kit (Takara Bio, Kusatsu, Japan).

### 2.3. Gene Cloning and Vector Construction

The full-length coding sequence (CDS) of *MaDIV* (Gene ID: ZS5801Monoploid.020078.1) was amplified from mulberry fruit cDNA using Phanta^®^ Max Super-Fidelity DNA Polymerase (Vazyme, P505-d3, Nanjing, China) with gene-specific primers (F: 5′-ATGTACAGTGGAATTGGGAT-3′; R: 5′-TCACTGATGATGCATGGATT-3′). The PCR product was cloned into the pEAZY^®^-Blunt Zero Cloning Vector (TransGen Biotech, CB501-01, Beijing, China) and verified by Sanger sequencing (Sangon Biotech, Shanghai, China). The verified CDS was then subcloned into the plant overexpression vector *pLGNL* (Bio SCI, Hangzhou, China) using *BamHI* and *SalI* restriction sites (Thermo Fisher Scientific) via T4 DNA Ligase (Takara Bio). For subcellular localization, the *MaDIV* CDS (without the stop codon) was fused in-frame to the 5′ end of the Green fluorescent protein (GFP) gene in the *pCAMBIA1300-GFP* vector (Bio SCI, Hangzhou, China) using *SacI* and *KpnI* sites.

### 2.4. Identification and Phylogenetic Analysis of MaMYB

To identify putative MYB transcription factors in the mulberry (*Morus atropurpurea*) genome of ZS5801 [23], we applied an integrated approach combining hidden Markov model (HMM) and BLAST-based searches. The HMM profiles for MYB DNA-binding domains (PF00249) were obtained from the InterPro [25]. First, the sequences were searched using hmmsearch from the HMMER suite (v3.3) with the domain-specific HMM profiles (E-value < 1 × 10^−5^). For further validation, a multiple sequence alignment of the candidate sequences was generated with MAFFT (v7.505), A refined HMM profile was constructed from this alignment using hmmbuild. This profile was then used for a second iteration of hmmsearch against the complete protein file to enhance detection sensitivity. Complementarily, a local BLASTP (v2.13.0+) database was constructed using 16,030 known MYB protein sequences obtained from the PlantTFDB [26]. All predicted protein sequences from the ZS5801 genome were then queried against this custom database using BLASTP (E-value threshold of 1 × 10^−10^). The final high-confidence MYB candidates were identified as the intersection of the HMM and BLAST result.

The AtMYB sequence of *Arabidopsis thaliana* was downloaded from PlantTFDB. Multiple sequence alignment was performed using MAFFT (v7.505) with the --maxiterate 1000 and -localpair options. The phylogenetic tree was reconstructed using IQ-TREE 2 (v2.2.0) [27] with the best-fit substitution model automatically selected via the ModelFinder (MFP) algorithm. Branch supports were assessed with 1000 ultrafast bootstrap replicates and 1000 SH-aLRT tests. Evolutionary trees are classified based on *Arabidopsis thaliana* [28]. The tree was further beautified by the iTOL website (https://itol.embl.de/, accessed on 28 January 2026) [29].

### 2.5. Characterization of Individual MYB Genes

MEME (http://meme-suite.org/tools/meme, 20 October 2025) was used to identify the conserved motifs among mulberry MaMYB proteins and motif information was visualized by Tbtools [30]. The 2000 bp upstream sequences of all *MaMYB* genes were extracted. Putative cis-regulatory elements in these sequences were predicted using the PlantCARE database (http://bioinformatics.psb.ugent.be/webtools/plantcare/html/); then the visualization was performed by TBtools (v.2. 458). The subcellular localization of these proteins was predicted using the DeepLoc online server [31].

To systematically characterize the biochemical properties of the candidate proteins, we calculated 12 key physicochemical parameters based on their amino acid sequences. Using the Biopython toolkit, all computations were performed solely at the level of the primary amino acid sequence, without considering the effects of post-translational modifications.

### 2.6. Quantitative Real-Time PCR (qRT-PCR) Analysis

Quantitative primers were designed using Primer Premier 5.0 (Premier Biosoft, Palo Alto, CA, USA). qRT-PCR was performed on a CFX96 Touch™ Real-Time PCR Detection System (Bio-Rad) using SYBR^®^ Premix Ex Taq™ II (Tli RNaseH Plus) (Takara Bio, RR820 A, Beijing, China). Each 20 μL reaction contained 10 μL of SYBR Premix, 0.4 μM of each primer, and 50 ng of cDNA template. The thermal cycling protocol was as follows: 95 °C for 30 s, followed by 40 cycles of 95 °C for 5 s and 60 °C for 30 s. A melt curve analysis was generated from 65 °C to 95 °C with 0.5 °C increments to confirm amplification specificity. The mulberry *MaRPL15* gene and tobacco *NtActin* gene were used as internal controls for normalization [32,33]. Gene-specific primers are listed in Supplementary Table S1. Relative expression levels were calculated using the 2^−ΔΔCt^ method. Three biological replicates and three technical replicates were analyzed for each sample.

### 2.7. Subcellular Localization

The MaDIV-GFP fusion construct and the empty *pCAMBIA1300-GFP* vector were separately transformed into *Agrobacterium tumefaciens* strain GV3101. The agrobacteria were cultured overnight at 28 °C in LB medium containing 50 μg/mL kanamycin and 50 μg/mL rifampicin, collected by centrifugation, and resuspended in infiltration buffer (10 mM MgCl_2_, 10 mM MES pH 5.6, 150 µM acetosyringone) to an OD_600_ of 0.6. The suspensions were infiltrated into the abaxial side of 4-week-old *Nicotiana benthamiana* leaves using a needleless syringe. After 48 h of incubation at 22 °C under a 16 h light/8 h dark photoperiod, leaf disks were examined using a confocal laser scanning microscope (Leica SP8) with excitation at 488 nm and emission at 500–530 nm for GFP, and at 405 nm and 450–500 nm for DAPI-stained nuclei. Images were processed using the microscope’s software.

### 2.8. Tobacco Transformation and Transgenic Line Validation

The transformation protocol was adapted from published literature with minor adjustments [34]. The recombinant *pLGNL-MaDIV* plasmid was introduced into *Agrobacterium tumefaciens* strain GV3101 (pSoup-P19) via the freeze–thaw method. Sterile leaf discs (1 cm^2^) were immersed in an Agrobacterium suspension (OD_600_ = 0.6–0.8) for 10 min, blotted dry, and co-cultivated on MS medium containing 200 μM acetosyringone in the dark at 25 °C for 48 h. Explants were then transferred to selection medium (MS salts, 3% sucrose, 0.8% agar, 1.0 mg/L 6-benzylaminopurine, 0.1 mg/L α-naphthaleneacetic acid) supplemented with 50 mg/L kanamycin and 300 mg/L cefotaxime and subcultured every 2 weeks. Regenerated shoots were transferred to rooting medium (1/2 MS salts, 0.1 mg/L indole-3-butyric acid). Resistant plantlets were rooted and acclimatized before being transferred to soil. Positive transgenic lines were confirmed by qRT-PCR analysis of *MaDIV* expression. Three independent transgenic lines with stable *MaDIV* expression were generated and analyzed.

### 2.9. Anthocyanin Content Measurement

Anthocyanin extraction and measurement were performed as described by Chen et al. [35] with minor modifications. Approximately 0.025 g of fresh leaf or petal tissue was ground to a fine powder in liquid nitrogen and homogenized in 500 μL of acidified methanol (methanol: hydrochloric acid = 95:5, *v*/*v*). The homogenate was incubated at 4 °C in darkness for 16 h with gentle shaking. After centrifugation at 12,000× *g* for 10 min at 4 °C, 200 μL of the supernatant was mixed with 200 μL of ultrapure water and 200 μL of chloroform. The mixture was vortexed and centrifuged at 12,000× *g* for 1 min. The absorbance of the upper aqueous phase was measured at 530 nm using a microplate reader (Sangon Biotech). Anthocyanin content was expressed as A_530_ per gram fresh weight (A_530_/g FW). Three biological replicates were measured for each sample.

### 2.10. Statistical Analyses

Statistical analyses were performed using IBM SPSS Statistics (Version 23.0) and GraphPad Prism (Version 8.0). For comparisons between two groups, a two-tailed Student’s *t*-test was used. For multiple group comparisons, a one-way analysis of variance (ANOVA) was performed, followed by Tukey’s post hoc test for pairwise comparisons. All experiments were performed with at least three independent biological replicates. Data are presented as mean ± standard deviation (SD). Asterisks indicate statistical significance: * < 0.05; ** < 0.01.

## 3. Results

### 3.1. Identification of MYB Family Genes in Mulberry

**Figure 1:**
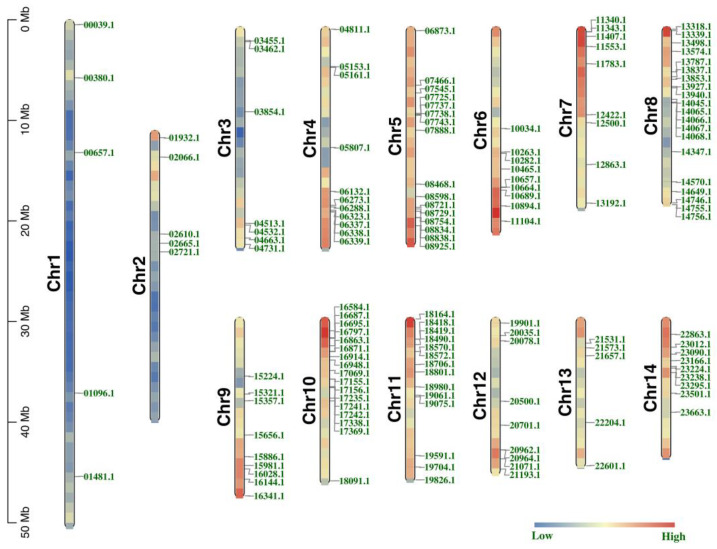
Chromosome mapping of 145 *MaMYBs*. The scale bar on the left indicates the length (Mb) of the chromosome. The colors on the chromosomes indicate gene density.

To elucidate the regulatory potential of MYB transcription factors in mulberry, a total of 145 *MaMYB* genes were identified based on genome-wide analysis. These genes were unevenly distributed across all 14 chromosomes of the mulberry genome, with chromosome 8 harboring the highest number (20 genes), while chromosomes 1, 2 and 13 contained the fewest (5 genes each) (Figure 1 and Figure S1). The 145 MaMYB proteins exhibited considerable diversity in length, molecular weight, and isoelectric point, with the majority predicted to localize to the nucleus (Table S2).

### 3.2. Phylogenetic and Gene Duplication Analysis of the MaMYB Proteins

**Figure 2:**
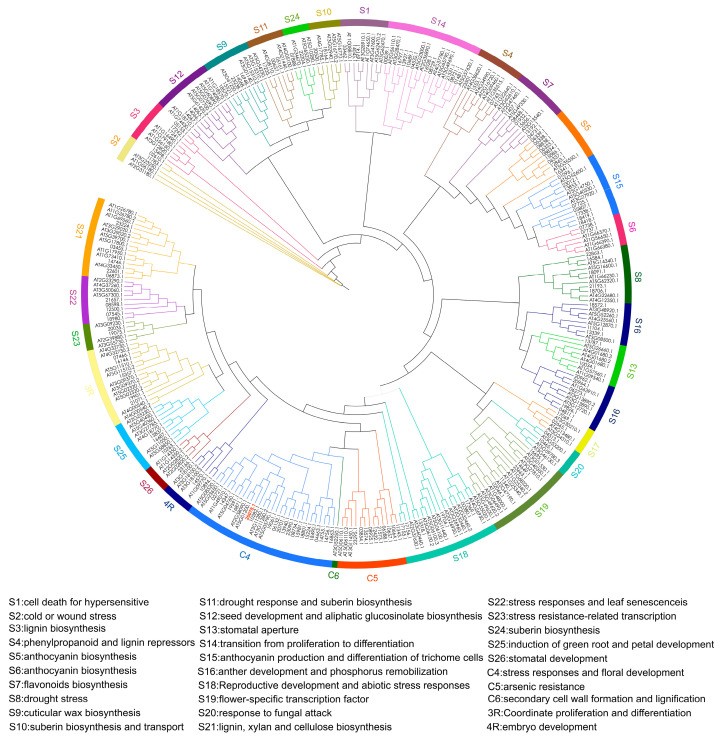
Phylogenetic analysis of MYB proteins in mulberry and *Arabidopsis thaliana*. The tree shows 31 phylogenetic clusters (S1–S26, C4–C6, 3R–4R), and different colors areas denote clusters. The legend below the phylogenetic tree describes the reported functions of genes in each *Arabidopsis* subfamily.

Phylogenetic analysis of the MaMYB proteins, together with their counterparts from *Arabidopsis thaliana*, classified them into 31 distinct subfamilies (Figure 2). Further divergence is evident in subfamilies such as C4, which is significantly expanded in mulberry (20 genes vs. 9 in Arabidopsis), and S5 (9 vs. 1). These asymmetrical distributions highlight distinct evolutionary trajectories, potentially driven by species-specific adaptive pressures.

Gene duplication events were identified using DupGen_finder with rice as the outgroup. A total of 19 tandem duplicate gene pairs were detected among mulberry MYB genes. *Ka*/*Ks* analysis indicated that these tandem duplicated gene pairs were under strong purifying selection, suggesting their highly conserved and functionally important roles (Figure S2, Table S3). Considering that no recent whole-genome duplication (WGD) event occurred in mulberry [23], we further performed BLASTP searches within the MaMYB family and conducted *Ka*/*Ks* analysis for dispersed duplicate gene pairs. The results showed that these duplicated genes were also mainly constrained by purifying selection, supporting that the MYB gene family is functionally conserved and essential during mulberry evolution (Table S4).

### 3.3. Conserved Motif and Gene Structure Analysis

**Figure 3:**
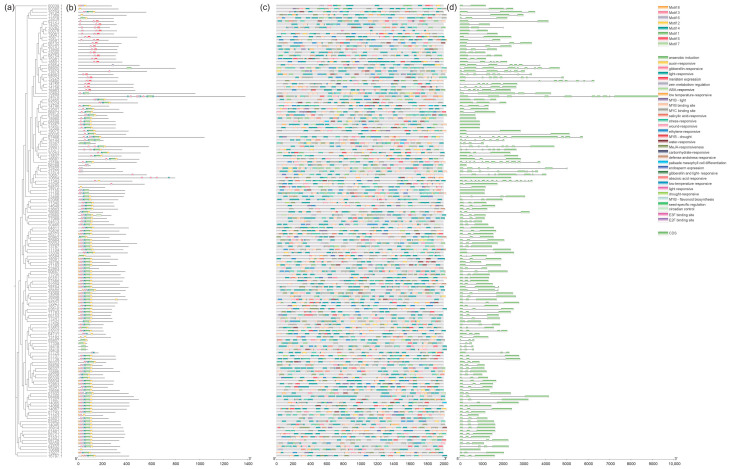
Phylogenetic relationship (**a**), conserved motif analyses (**b**), predicted cis-elements (**c**) and gene structure (**d**) of MaMYB genes in mulberry. Different motifs are represented by various colored boxes and numbers (1–8). The boxes of different colors on the promoters represent different cis-elements. The green box represents the CDS, the black lines indicate the intron. The scale bar is shown at the bottom.

Conserved motif and gene structure analyses further supported the phylogenetic classification. Proteins within the same subgroup shared similar motif compositions and exon-intron architectures (Figure 3). We identified a total of eight motifs, named motif 1 to motif 8. The MaMYB protein motifs located in the same subfamilies were similar in class and order, whereas the number of motifs in the different groups varied considerably. Using TBtools [30], we generated sequence logos of all identified MaMYB motifs, which were predominantly located in the N-terminal region of most MaMYB proteins (Figure 3b and Figure S3a).

Analysis of *cis*-acting regulatory elements in the promoter regions of MaMYB genes revealed a diverse array of functional elements, underscoring their involvement in complex regulatory networks (Figure 3c). Light-responsive elements were the most abundant (1300, 17.37%), highlighting the potential significance of light signaling in modulating *MaMYB* expression. Substantial numbers of hormone-responsive elements were also identified, including those for Abscisic acid (ABA, 683, 9.13%), ethylene (185, 2.47%), jasmonic acid (187, 2.50%), and gibberellin (125, 1.67%). Furthermore, numerous elements associated with stress responses (e.g., low temperature: 68, 0.91%; wound: 161, 2.15%; stress: 339, 4.53%) and key binding sites for MYB (835, 11.16%) and MYC (446, 5.96%) transcription factors were prevalent, suggesting roles in abiotic stress adaptation and transcriptional cascades (Figure S3b).

The gene structure results showed that the number of exons and introns in the *MaMYB* gene family varied considerably, ranging from 1 to 16. Ten *MaMYB* genes had only one exon and no introns. In total, 143 *MaMYB* genes contained 1–10 exon, and only 2 *MaMYB* genes contained 10–20 exons. 011343.1 has the highest number of exons (16) (Figure 3d). Consistent exon-intron structure patterns were observed among members of the same subgroup, particularly regarding exon number and length, suggesting a degree of conservation (Figure S3c).

### 3.4. MaDIV Is Negatively Correlated with Mulberry Anthocyanins

**Figure 4:**
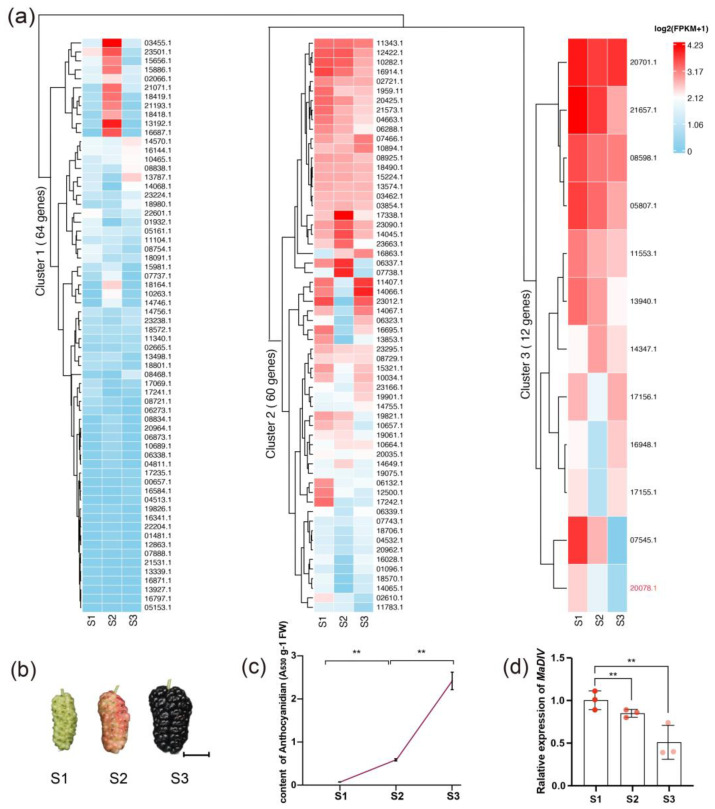
MaDIV is negatively correlated with mulberry anthocyanins. (**a**) Expression profiling of differentially expressed *MaMYB* genes. S1: green stage of mulberry fruit; S2: color-turning stage of mulberry fruit; S3: ripe fruit stage of mulberry fruit, the same applies below. Genes highlighted in red denote *MaDIV* (*20078.1*). (**b**) Mulberry fruit phenotype of ZS5801 at different developmental stages. Scale bar: 1 cm. (**c**) Anthocyanin content at different developmental stages of ZS5801 fruits. (**d**) qRT-PCR results of *MaDIV* gene expression at three developmental stages of mulberry fruit, with expression levels normalized to the S1 stage. The ** in the graph indicates *p* < 0.01 between the two groups, the same applies below.

To investigate the expression patterns of *MaMYBs* in mulberry fruits, Fragments per kilobase of transcript per million mapped reads (FPKM) values of *MaMYB* genes across three developmental stages of ZS5801 mulberry fruits were acquired from the transcriptome data (Figure 4a). The expression of most *MaMYB* genes differed significantly across stages. After excluding genes not expressed throughout all developmental stages, 136 *MaMYB* genes remained. Different expression patterns highlight the temporal specialization of *MaMYB* regulators and provide putative genes for further functional studies on fruit development and ripening in mulberry.

The phenotype of fruits of the ZS5801 mulberry undergoes significant changes during S1 to S3 (Figure 4b). Anthocyanin levels exhibited substantial accumulation, increasing by 750.7% at S2 and 3414.5% at S3 relative to S1. At S3, anthocyanin concentrations reached 2.43 A_530_/g fresh weight (FW) in mulberry fruit (Figure 4c). Among the 145 MaMYB genes identified, we focused on those exhibiting a progressive decline in expression during fruit ripening, as this pattern is consistent with a potential repressive role in anthocyanin accumulation. Applying a threshold of S1 > 50 and S3 < 10 (FPKM), two candidates remained: *07545.1* and *20078.1* (MaDIV) (Table S5). Among the two candidates, *07545.1* is a putative ortholog of *AtMYBR1/44,* a well-characterized anthocyanin repressor [36,37,38]. All *Ka*/*Ks* values for gene pairs involving *07545.1* in ZS5801 are far below 1, indicating that these genes are under strong purifying selection and have retained conserved functions (Table S4). By contrast, *MaDIV* belongs to the DIVARICATA-like subfamily, a group with emerging but poorly characterized roles in specialized metabolism, and its potential involvement in anthocyanin regulation has not been previously explored. Based on this distinction, we prioritized *MaDIV* to investigate whether DIV-like MYB transcription factors play a previously unrecognized role in anthocyanin repression. Consistent with the transcriptomic data, the expression level of *MaDIV* decreased by 15.28% at the S2 stage and by 49.17% at the S3 stage, relative to the S1 stage (Figure 4d).

**Figure 5:**
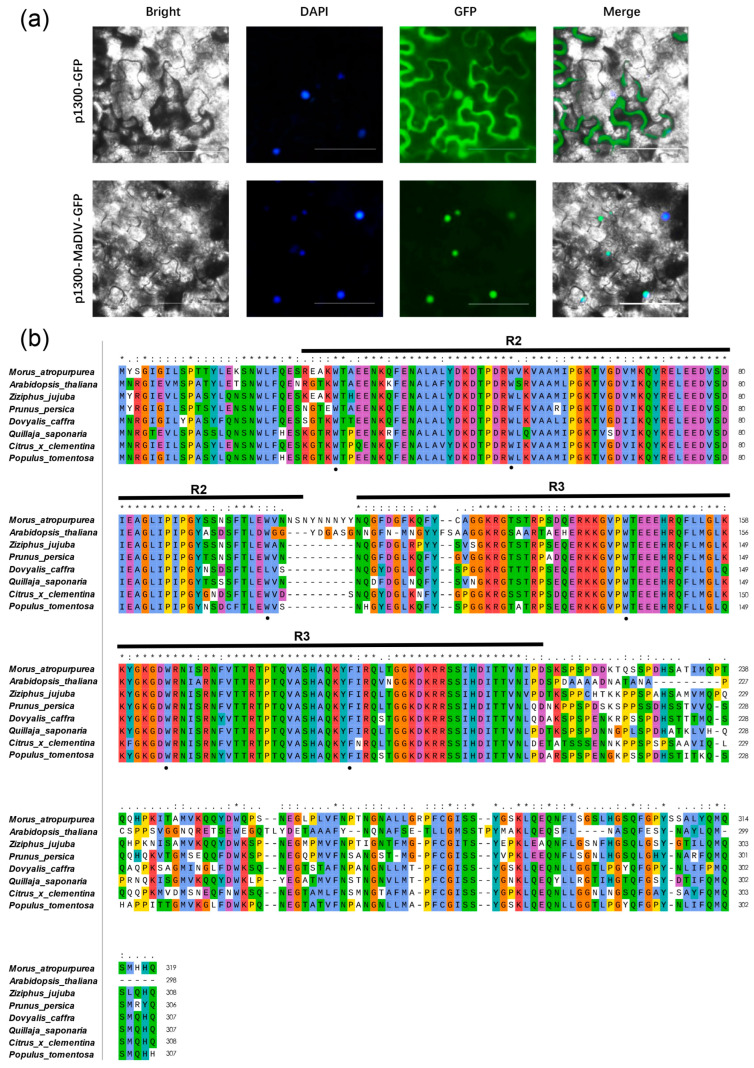
Subcellular location and protein sequence alignment of MaDIV. (**a**) Subcellular location of MaDIV protein. The images depict bright field, blue fluorescence of the DAPI-labeled nucleus, GFP fluorescence, and merged of *p1300*-GFP and *p1300*-MaDIV-GFP. The scale bars measure 100 μm. (**b**) Protein sequence alignment of DIV in various species. The R2 and R3 domains are denoted by black lines above the protein sequence, while the conserved tryptophan (Trp, W) residues characteristic of the DIV subfamily are indicated by black dots below the sequence. Colors follow the standard Clustal X scheme for amino acid properties. Conservation symbols: * = identical residues in all sequences; : = conserved substitutions; . = semi-conserved substitutions; no symbol = divergent positions.

Consistent with its predicted function as a transcription factor, bioinformatic prediction indicated that MaDIV is likely a nuclear-localized protein (Table S2). To determine the subcellular localization of MaDIV, we expressed a MaDIV-GFP fusion protein in *Nicotiana benthamiana* leaves. Confocal microscopy revealed that the MaDIV-GFP signal was localized exclusively to the nucleus (Figure 5a). MaDIV, along with its closest orthologs, possesses a unique combination of motifs, including motif 3 and motif 5, which are characteristic of the DIV-like subfamily and may be associated with its repressor function. Gene structure analysis indicated that MaDIV contains two exons and one intron, an architecture conserved among its orthologs (Figure 3). Phylogenetic analysis revealed that MaDIV clusters within the DIVARICATA clade of R2R3-MYB transcription factors, showing the closest relationship with the ortholog from AT5G01200.1, AT2G38090.1, AT5G08520.1, AT5G23650.1, AT1G49010.1, AT5G04760.1, AT3G11280.1/2 and AT5G05790.1, belong to C4 subfamily (Figure 2).

Homologs of MaDIV from *Morus atropurpurea* were identified in *Arabidopsis thaliana*, *Ziziphus jujuba*, *Prunus persica*, *Dovyalis caffra*, *Quillaja saponaria*, *Citrus* x *clementina*, and *Populus tomentosa*, all of which possess the conserved R2 and R3 domains (Figure 5b). The analysis revealed that the conserved tryptophan (Trp, W) residues are spaced approximately 18 amino acids apart. The R2 domain contains three Trp residues. In contrast, only the first and second Trp residues are conserved in the R3 domain, with the third Trp consistently replaced by phenylalanine (F).

### 3.5. Overexpression of MaDIV in Tobacco Is Associated with Anthocyanin Biosynthesis

**Figure 6:**
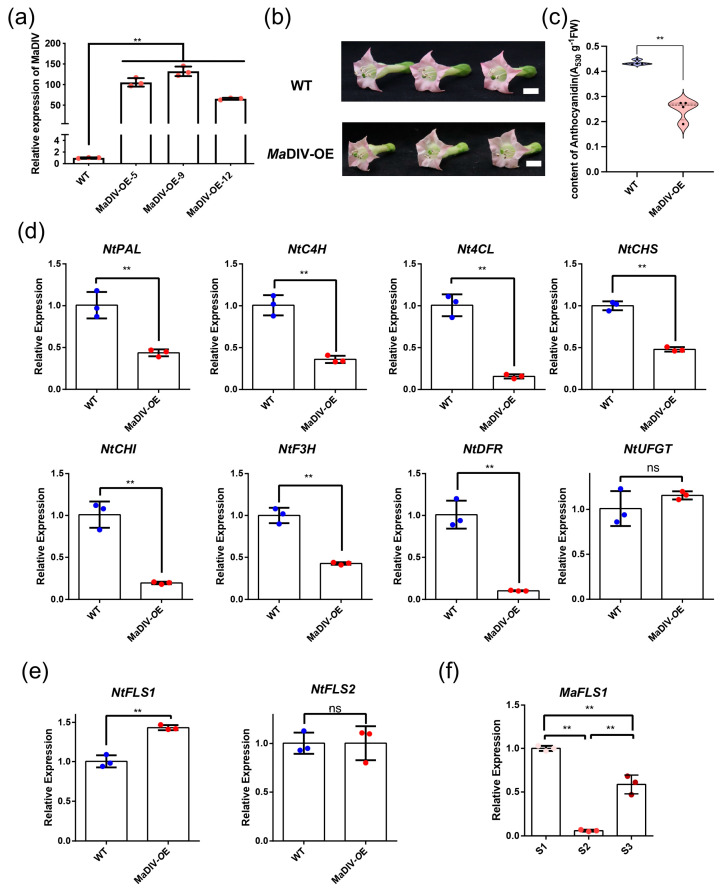
*MaDIV* is associated with changes in anthocyanin accumulation. (**a**) Validation of overexpression in tobacco. (**b**) Phenotype of the overexpression tobacco flowers; scale bar: 1 cm. (**c**) Anthocyanin content in the flowers shown in panel (**b**). (**d**) Expression levels of anthocyanin biosynthetic genes in *MaDIV*-overexpressing tobacco. (**e**) Expression of two *NtFLS* genes in *MaDIV*-overexpressing tobacco. (**f**) Expression patterns of *MaFLS1* during different developmental stages of mulberry fruit. In the graph, ** indicates *p* < 0.01 between the two groups, and “ns” indicates *p* > 0.05 (no significant difference).

Following the successful generation of transgenic tobacco lines constitutively overexpressing the *MaDIV* gene (Figure 6a), a pronounced alteration in floral pigmentation was observed (Figure 6b). Quantitative analysis revealed that the anthocyanin content in the transgenic flowers was significantly reduced to 0.249 A_530_/g FW, representing a 42% decrease compared to the wild-type (WT) plants (Figure 6c).

Subsequent expression profiling of core anthocyanin biosynthetic genes demonstrated a marked downregulation of *NtPAL*, *NtC4H*, *Nt4CL*, *NtCHS*, *NtCHI*, *NtF3H* and *NtDFR* (Figure 6d). The transcript levels of two major *flavonol synthase* (*NtFLS*) genes in tobacco plants overexpressing *MaDIV* were examined. The results showed that *NtFLS1* expression was significantly upregulated concomitant with the elevated expression of *MaDIV*, whereas no significant difference was observed in *NtFLS2* expression (Figure 6e). To further explore this observation, we analyzed the expression patterns of *MaFLS* genes during different developmental stages of mulberry fruit. The results showed a gradual decrease in *MaFLS* expression as the fruit ripened, a trend that paralleled the expression pattern of *MaDIV* and inversely correlated with anthocyanin accumulation (Figure 6f).

## 4. Discussion

The precise regulation of anthocyanin biosynthesis represents a fundamental aspect of plant secondary metabolism [39]. This study suggests that *MaDIV*, a R2R3-MYB transcription factor in mulberry, may be involved in the regulation of anthocyanin biosynthesis, although direct evidence such as promoter binding assays is required to confirm this role. These findings reveal a novel regulator in a commercially significant plant and substantially broaden the functional understanding of the DIV MYB subfamily beyond its established roles in developmental processes. The selection of *MaDIV* from a genome-wide screen highlights the value of integrating phylogenetic classification with expression profiling to identify non-canonical regulators. This approach enabled the identification of a DIV-like MYB with previously unrecognized repressive activity in the anthocyanin pathway.

Genome-wide analysis revealed 145 *MYB* genes in *Morus atropurpurea*, a notable expansion compared to the 103 members reported in *M. alba*. [6], which further enriches the understanding of this family in the *Morus* genus. This discrepancy may reflect lineage-specific adaptations following the divergence of the two species. From an evolutionary perspective, the expansion and diversification of the MYB family in mulberry highlight the adaptive evolution of transcriptional regulators in perennial fruits. The DIV-like subfamily, to which MaDIV belongs, exhibits conserved motif architectures and exon-intron structures, implying functional conservation across plants. The presence of abundant light-, hormone-, and stress-responsive cis-elements in the promoters of *MaMYB* genes suggests their integration into broader signaling networks, enabling plants to modulate pigment accumulation in response to environmental cues. From the subset of *MYB* genes whose expression declined during fruit ripening, two strong candidates emerged: *07545.1* and *MaDIV* (*20078.1*). Duplication analysis revealed that *MaDIV* exists as a singleton in the mulberry genome, with no evidence of recent tandem or whole-genome duplication events. In contrast, *07545.1* belongs to a dispersed duplication cluster under strong purifying selection (*Ka*/*Ks* < 0.3). Given that *07545.1* is a putative ortholog of the well-characterized repressor *AtMYBR1/44*, its function is largely predictable. By contrast, MaDIV belongs to the DIV-like subfamily, a group with ill-defined roles in anthocyanin regulation, providing a compelling rationale for prioritizing *MaDIV* for functional analysis.

MYB transcription factors are well-known regulators of the phenylpropanoid pathway, with particular importance in controlling anthocyanin accumulation [40,41,42,43]. These proteins function as either activators or repressors within the MYB-bHLH-WD40 complex to modulate pigmentation in response to developmental and environmental signals [44]. While R2R3-MYB activators in anthocyanin accumulation such as *GmMYBA* in soybean and *StMYB210* in potato tuber flesh have been extensively characterized [45,46], the repressive members, especially those belonging to the DIV-like clade, remain inadequately explored. Phylogenetic analysis positioned MaDIV within the evolutionarily distinct DIVARICATA clade of the C4 subfamily. The C4 clade gene *At5g04760* in *Arabidopsis thaliana* encodes *AtDIV2*, the functional ortholog of *CmaCh15G005270*, a candidate gene responsible for the seed coat color phenotype produced by the proanthocyanidins in *Cucurbita maxima* [47,48]. Our results provide further evidence suggesting that *MaDIV* may be associated with anthocyanin accumulation, thereby broadening the functional scope of this evolutionarily conserved subfamily.

During mulberry fruit ripening, anthocyanin accumulation is tightly coordinated with the expression of regulatory MYB protein. Previous studies have shown that R2R3-MaMYBs with late-expression patterns, such as *MaMYB43*, *77*, *83*, *98*, and the characterized activator *MaMYB58* (*MaMYBA*), are positively correlated with anthocyanin accumulation [6,49]. Conversely, we observed a marked decline in *MaDIV* transcript levels as pigmentation intensified, suggesting a potential negative correlation between its expression and anthocyanin content. This expression pattern parallels those observed for other documented repressive *MYBs*, such as *FaMYB6* in strawberry and *VvMYB24* in grape [50,51], supporting a potential role for *MaDIV* in preventing premature pigment deposition during early fruit development, consistent with the spatial brake model of anthocyanin regulation. The concomitant downregulation of *MaDIV* and upregulation of activators such as *MaMYBA* during ripening suggests a coordinated regulatory module that fine-tunes anthocyanin biosynthesis in a stage-specific manner.

Heterologous overexpression of *MaDIV* in tobacco supported its potential function. Transgenic plants exhibited severely reduced floral pigmentation accompanied by coordinated downregulation of multiple anthocyanin biosynthetic genes, consistent with the behavior of established R2R3-MYB repressors such as *PhMYB27* in petunia and *PtrMYB182* in poplar [52,53]. The expression of *NtDFR*, which encodes a key enzyme converting (+)-dihydroflavonols to (+)-leucoanthocyanidins in the anthocyanin pathway, was strongly suppressed, indicating a restriction at this enzymatic bottleneck in anthocyanin biosynthesis [54]. Concurrently, *MaDIV* overexpression appeared to upregulate *NtFLS1* specifically without altering *NtFLS2* expression, though further research is needed to prove direct binding of *MaDIV* to the promoters of these genes. The upregulation of *NtFLS1* in *MaDIV*-overexpressing tobacco, together with the downregulation of *DFR*, raises the possibility of a shift in flavonoid metabolic flux, although direct evidence such as flavonol measurement is required to support this hypothesis. The parallel expression patterns of *MaDIV* and *MaFLS* during mulberry fruit ripening are consistent with this possibility. DFR and FLS compete for (+)-dihydroflavonol substrates to produce anthocyanins and flavonols, respectively [55,56]. However, whether this reflects a direct regulatory mechanism or an indirect consequence remains to be investigated. Therefore, the proposed metabolic shift, in the absence of flavonol quantification or metabolomic data, requires direct experimental validation.

It is important to acknowledge the limitations of using a heterologous expression system. While tobacco is a well-established model for preliminary functional studies of plant transcription factors, it may not fully recapitulate the native regulatory environment of mulberry. Pending complementary loss-of-function studies in mulberry, we refer to *MaDIV* as a candidate regulator throughout the manuscript. Current evidence is largely correlative, and the precise molecular mechanism by which *MaDIV* exerts its repressive effect remains to be elucidated. Future studies should employ yeast one-hybrid assays, dual-luciferase reporter systems, competitive assays, EMSA, or ChIP-qPCR to determine whether *MaDIV* directly binds to the promoters of key anthocyanin biosynthetic genes, particularly *DFR* or *FLS*. Additionally, the proposed redirection of metabolic flux toward flavonol biosynthesis is based solely on gene expression data without direct measurement of flavonol levels or metabolomic profiling. Metabolomic analyses such as flavonol quantification would help clarify whether the observed gene expression changes translate into altered metabolic flux. Such experiments will be essential to fully elucidate the molecular mechanism by which *MaDIV* exerts its potential role. Therefore, the proposed regulatory model should be considered a hypothesis that requires experimental validation.

## 5. Conclusions

In conclusion, the genome-wide analysis identified 145 MaMYB genes classified into 31 subfamilies, and functional characterization of *MaDIV* suggests its possible involvement in anthocyanin accumulation in mulberry. Our work provides a foundation for further investigation of DIV-like MYB transcription factors in fruit crops.

## Data Availability

The original experimental data available on request from the authors. The genome and transcriptome data of mulberry cultivar ZS5801 are derived from a publicly available repository [23].

## Supplementary Materials

The following supporting information can be downloaded at: https://www.mdpi.com/article/10.3390/genes17060702/s1, Figure S1: The number of *MYB* genes on each chromosome; Figure S2: Duplication gene pairs of *MaMYBs* in the mulberry genome; Figure S3: The statistics of the number of core sequence logos (**a**), cis-elements with the promoter (**b**) and exons combined (**c**); Table S1: The primer sequences used for qRT-PCR; Table S2: Protein physicochemical properties; Table S3: Tandem duplicate gene pairs; Table S4: Dispersed duplicate gene pairs; Table S5: The MYB genes negatively correlated with anthocyanin accumulation throughout mulberry fruit developmental stages.

## Abbreviations

The following abbreviations are used in this manuscript:

ABA	Abscisic acid
ANOVA	Analysis of variance
ANS	Anthocyanidin synthase
CDS	Coding sequence
CHI	Chalcone isomerase
CHS	Chalcone synthase
C4H	Cinnamate 4-hydroxylase
DFR	Dihydroflavonol 4-reductase
DIV	DIVARICATA
F3H	Flavanone 3-hydroxylase
FLS	Flavonol synthase
FPKM	Fragments per kilobase of transcript per million mapped reads
GFP	Green fluorescent protein
GRAVY	Grand Average of Hydropathicity
HMM	Hidden Markov Model
MBW	MYB-bHLH-WD40 complex
MS	Murashige and Skoog medium
MYB	Myeloblastosis
PAL	Phenylalanine ammonia-lyase
qRT-PCR	Quantitative real-time polymerase chain reaction
TF	Transcription factor
WT	Wild-type
