# Supplementary figures and images for: Genome-Wide Identification of the MYB Family in Morus atropurpurea and Functional Characterization of MaDIV for Its Possible Involvement in Anthocyanin Biosynthesis
Source: Genes (Basel). 2026 Jun 17;17(6):702. doi: 10.3390/genes17060702 (PMC13300443; doi:10.3390/genes17060702)

# Chromosomes of Mulberry

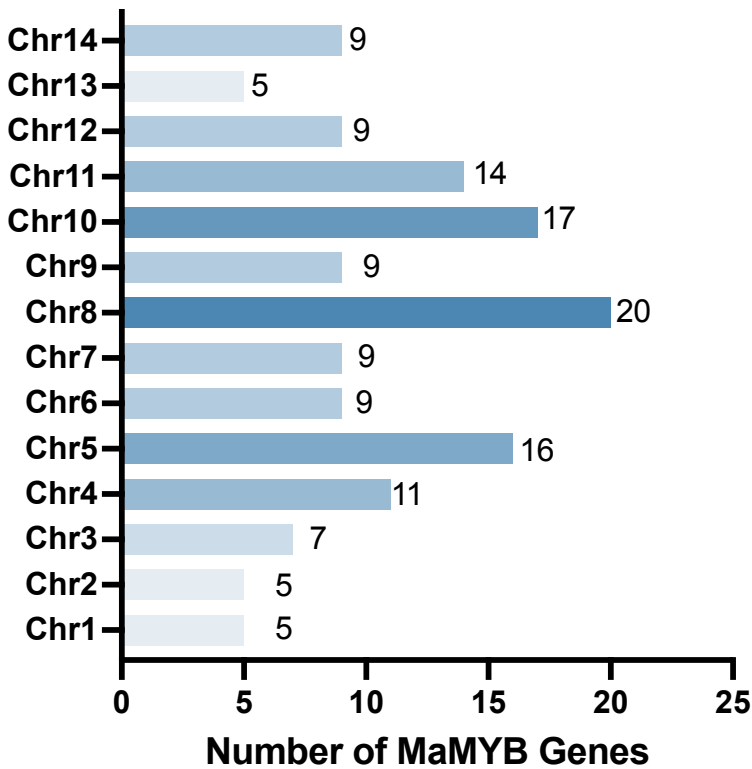

Supplement: Supplementary file 1 [file genes-17-00702-s001.zip › Fig S1.pdf]

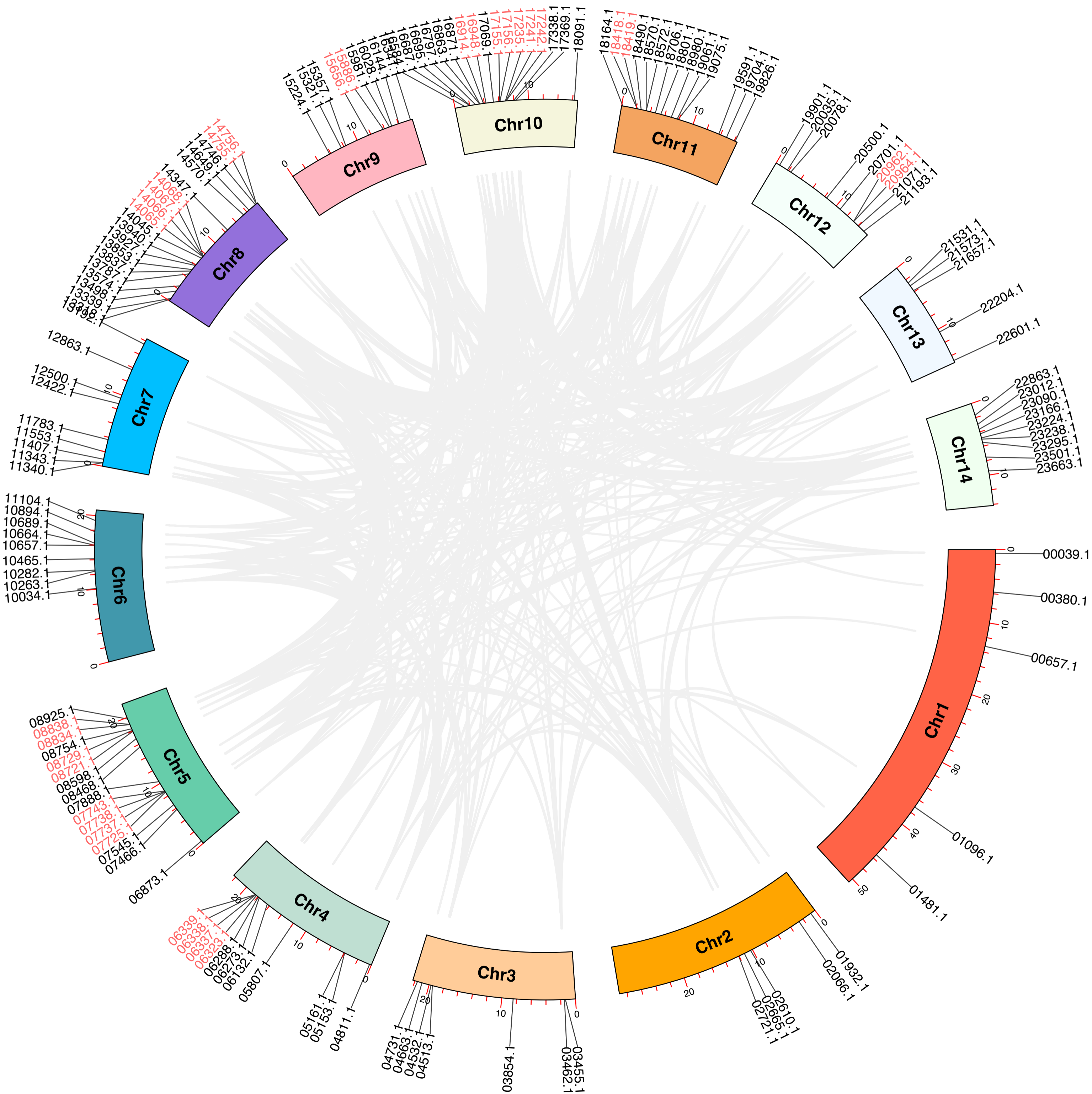

Supplement: Supplementary file 1 [file genes-17-00702-s001.zip › Fig S2.pdf]

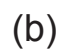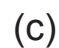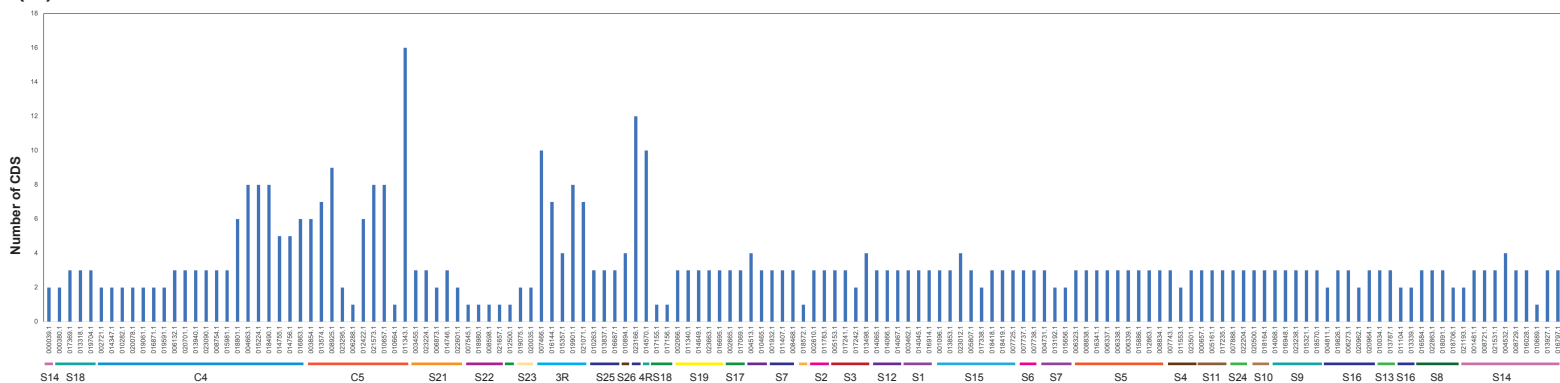

Supplement: Supplementary file 1 [file genes-17-00702-s001.zip › Fig S3.pdf]
